# Workaholism among *stricto sensu* graduate nursing professors in Brazil*

**DOI:** 10.1590/1518-8345.4071.3326

**Published:** 2020-08-31

**Authors:** Laio Preslis Brando Matos de Almeida, Maynara Fernanda Carvalho Barreto, Júlia Trevisan Martins, Maria do Carmo Fernandez Lourenço Haddad, Maria José Quina Galdino

**Affiliations:** 1 Universidade Estadual do Norte do Paraná, Bandeirantes, PR, Brazil.; 2 Universidade Estadual de Londrina, Londrina, PR, Brazil.; 3 Universidade Estadual de Maringá, Maringá, PR, Brazil.

**Keywords:** Occupational Health, Education, Nursing, Graduate, Faculty, Universities, Work Performance, Working Conditions, Saúde do Trabalhador, Educação de Pós-Graduação em Enfermagem, Docentes, Universidades, Desempenho Profissional, Condições de Trabalho, Salud Laboral, Educación de Postgrado en Enfermería, Docentes, Universidades, Rendimiento Laboral, Condiciones de Trabajo

## Abstract

**Objective:**

to identify the prevalence and factors associated with workaholism among *stricto sensu* graduate nursing professors.

**Method:**

a cross-sectional study with 333 professors of master’s/doctorate degrees from 47 Brazilian public universities. Participants answered a characterization questionnaire and the Dutch Work Addiction Scale, which were analyzed descriptively and by multiple logistic regression.

**Results:**

the prevalence of workaholism was 10.5%. The factors associated with the dimensions of workaholism were: having a marital relationship, being dissatisfied with work and sleep, indicating low ability to concentrate and few leisure opportunities, belonging to Graduate Programs with grades 3, 4 and 5, receiving a research productivity grant, considering the influence of work on life as negative, showing difficulty in combining work with personal life, to present work-related anxiety, feel pressure for scientific publishing, elaborate more than 11 articles simultaneously, give more than 21 opinions in the last year, work an extra 11 hours a week in addition to the work schedule and dedicate less than 10 hours a week to graduate school.

**Conclusion:**

there is an indication of workaholism in the investigated professors, and the associated factors were related to working conditions and requirements. Universities must adhere to management models that include occupational health promotion.

## Abstract (pt)

**Objetivo:**

identificar a prevalência e os fatores associados ao *workaholism* entre docentes de pós-graduação *stricto sensu* em enfermagem.

**Método:**

estudo transversal com 333 docentes enfermeiros de mestrado/doutorado de 47 universidades públicas brasileiras. Os participantes responderam a um questionário de caracterização e a *Dutch Work Addiction Scale,* que foram analisados descritivamente e por regressão logística múltipla.

**Resultados:**

a prevalência de *workaholism* foi de 10,5%. Os fatores associados às dimensões do *workaholism* foram: possuir relacionamento conjugal, estar insatisfeito com trabalho e sono, indicar baixa capacidade de concentração e poucas oportunidades de lazer, pertencer aos Programas de Pós-graduação com conceitos 3, 4 e 5, receber bolsa produtividade em pesquisa, considerar a influência negativa do labor sobre a vida, mostrar dificuldade de compatibilizar trabalho com vida pessoal, apresentar ansiedade laboral, sentir pressão para realizar publicação científica, elaborar mais de 11 artigos concomitantemente, emitir mais de 21 pareceres no último ano, desenvolver mais de 11 horas semanais além do regime de trabalho e dedicar menos de 10 horas semanais à pós-graduação.

**Conclusão:**

há indicativo de *workaholism* nos docentes investigados e os fatores associados estiveram relacionados às condições e exigências laborais. As universidades devem aderir aos modelos gerenciais que englobem a promoção de saúde ocupacional.

## Abstract (es)

**Objetivo:**

identificar la prevalencia y los factores asociados con el *workaholism* entre los docentes de posgrado *stricto sensu* en enfermería.

**Método:**

estudio transversal con 333 docentes de maestría/doctorado de 47 universidades públicas brasileñas. Los participantes respondieron un cuestionario de caracterización y la *Dutch Work Addiction Scale* que se analizaron descriptivamente y mediante regresión logística múltiple.

**Resultados:**

la prevalencia de adicción al trabajo fue del 10,5%. Los factores asociados con las dimensiones de la adicción al trabajo fueron: tener una relación matrimonial, estar insatisfecho con el trabajo y el sueño, lo que indica una baja capacidad de concentración y pocas oportunidades de ocio, pertenecer a los programas de posgrado con los conceptos 3, 4 y 5, recibir una subvención de productividad, considerar la influencia negativa del trabajo en la vida, mostrar dificultades para combinar el trabajo con la vida personal, experimentar ansiedad laboral, sentir presión para realizar publicaciones científicas, elaborar más de 11 artículos simultáneamente, emitir más de 21 opiniones en el último año, desarrollar más de 11 horas a la semana además del régimen de trabajo y dedicar menos de 10 horas a la semana al posgrado.

**Conclusión:**

hay una indicación de adicción al trabajo en los docentes investigados y los factores asociados estaban relacionados con las condiciones y requisitos de trabajo. Las universidades deben adherirse a los modelos de gestión que abarcan la promoción de la salud ocupacional.

## Introduction

Studies have reported workaholism as one of the causes of workers’ mental and physical illness^(^ 1 ^-^ 3 ^)^. It is estimated that 10% of North Americans are workaholic^(^ 4 ^)^, this condition being more prevalent among workers who perform activities at the managerial level and specific sectors, such as health professionals and teachers^(^ 5 ^)^. Among nurses, a study carried out in Norway showed that this phenomenon is strongly associated with work characteristics rather than individual^(^ 6 ^)^.

The workaholic was, for a long time, considered as proactive and, therefore, a person’s quality, mainly in the industrial and business sector, which aims at high publishing as a result of the work process^(^ 7 ^)^. However, the term workaholism originates from the junction of the prefix “work” with the suffix “aholism”, to describe the addiction to work, characterized by compulsive and excessive work^(^ 8 ^)^, which is performed excessively and irrationally, as that even aware of the excess, the worker does not take control of the burden^(^ 9 ^)^.

In the contemporary world, it is clear that the organizational culture of the institutions is not far from this reality, including in universities, as it is observed in the dynamics of the teacher’s work, more specifically, in the Graduate Programs (*Programas de Pós-graduação*, PPG), some potential determinants for the development of workaholism.

In this sense, to promote technical and scientific progress, professors of the Graduate Nursing Programs (*Programas de Pós-graduação em Enfermagem*, PPGEnf) must daily perform teaching, research, extension and administrative activities, such as teaching classes at different levels of education; guide and evaluate monographs, dissertations, and theses, in addition to scientific initiation, extension, and teaching; coordinate teaching, research and extension projects; writing articles, editorials, books, chapters, opinions for scientific journals and funding agencies; organize and participate in national and international events and institutional working groups; coordinate courses and management and work commissions at universities, among others.

Most of these activities are taken into account in the PPGEnf evaluations by the Coordination for Personal Improvement in Higher Education (*Coordenação de Aperfeiçoamento Pessoal de Ensino Superior*, CAPES)^(^ 10 ^)^, which can lead the professor to work compulsively and excessively, dedicating substantial time and effort and giving up other activities such as leisure, family and friends, in addition to experiencing an environment of high competitiveness and high demand for publishing^(^ 11 ^)^. These elements increase the number of hours dedicated to work, in addition to the contract with the institution, which is fostered by information and communication technologies, making the worker not to detach from work, even outside away from it, leading to illness^(^ 7 ^)^.

Despite the above, few studies have identified the prevalence and factors associated with workaholism among individuals inserted in the work process of the different fields of activity in the public or private sector^(^ 1 ^-^ 3 ^)^, especially among nursing professors from PPGEnf. Studies that fill this gap are essential to support management actions and public policies about the risks to workers’ health since workaholism is a pathological condition that implies a decrease in professional performance, presenteeism^(^ 12 ^)^, techno-stress^(^ 7 ^)^, burnout^(^ 13 ^)^, deterioration of interpersonal relationships, mainly social and family relationships^(^ 2 ^)^, increased cardiovascular risk^(^ 14 ^)^ and *karoshi*, the sudden death from overwork^(^ 15 ^)^.

Therefore, the aim of this study was to identify the prevalence and factors associated with workaholism among *stricto sensu* graduate nursing professors.

## Method

Cross-sectional study conducted with PPGEnf professors from 47 public universities in the five regions of Brazil. These are programs recommended and recognized by CAPES, with grades 3 to 7, linked to Area 20 - Nursing, which offer master’s and/or academic doctoral courses.

The study population consisted of 919 permanent teachers accredited to the PPGEnf under study, according to data extracted from the Sucupira Platform in May 2018. Teachers with a degree in nursing and affiliated with the program for at least one year were included. Those who were on work leave were excluded. All professors were invited, and 368 answered the questionnaire, of which 333 met the study’s eligibility criteria and made up the sample of this investigation.

Data collection took place through a virtual platform, developed by two programmers, with training in Information Systems. JavaScript and Hypertext Preprocessor (PHP) programming languages were used for the frontend and backend of the system, in addition to the My Structured Query Language (MySQL) database management system. This platform housed the study instruments and was configured to send invitations to registered teachers every two weeks by e-mail, which contained the website that directed to the online questionnaire and was preceded by the acceptance to participate in the research through the Informed Consent Term.

From July to December 2018, participants answered a questionnaire prepared by the authors of sociodemographic characterization, living and working conditions with specific elements of the teaching work process: sex (male or female), marital status (with or without domestic relationship), Brazilian region to which PPGEnf belongs (North, Northeast, Midwest, Southeast or South), work arrangement (exclusive dedication, full time, part time or senior teacher - they retired and returned to work voluntarily in the PPGEnf), receiving a publishing grant (yes or no), number of accreditation PPG (one, two or three), working time in PPG (in years), teaching time (in years), weekly hours dedicated to postgraduate studies (in hours), hours in addition to the work contract on nights or weekends (in hours), level of performance at PPGEnf (master and doctorate, only masters or doctorate only), CAPES grades of PPGEnf (three, four, five, six and seven), graduate students under orientation (discrete number), articles under preparation (discrete number), opinions issued in the last 12 months for journals or funding agencies (discrete number), satisfaction with the work at PPGEnf (dissatisfied or satisfied), negative influence of the pace and intensity of work on his/her life (yes or no), demand for scientific publishing (yes or no), anxiety in the development of work activities (yes or no), difficulty in reconciling work with personal and family life (yes or no), memory ability and concentration (little or enough), leisure opportunities (little or enough) and satisfaction with sleep and rest (dissatisfied or satisfied).

The Dutch Work Addiction Scale (DUWAS) in its version adapted and validated for Brazilian Portuguese^(^ 16 ^-^ 17 ^)^ was used to evaluate workaholism in its two dimensions: compulsive work (items 1, 2, 4, 6 and 8) and excessive work (items 3, 5, 7, 9 and 10). The 10 items have 5-point Likert responses (0- never, and 4- always). The scores of these dimensions are dichotomized into high and low, considering the 75^th^ percentile as the cutoff point^(^ 2 ^,^ 17 ^)^, which after combined, emerge four different work patterns: positive or relaxed workers (people with low scores on overwork and work compulsive workers), hard workers (those who score high only on overwork), compulsive workers (high score only on compulsive work) and workaholics (high score on overwork and compulsive work).

Data were analyzed in the Statistical Package of Social Sciences (SPSS), version 20.0, by absolute, relative frequencies, medians and interquartile ranges (IIQ) (p-25-p75), since the quantitative variables did not adhere to the normal distribution, as indicated in the Shapiro-Wilk test (p<0.001). As all items were mandatory, there was no missing data.

The outcomes of this study were the two dimensions of workaholism: compulsive work and overwork. The associated factors were determined by multiple logistic regression models, which started with univariate analyzes, to verify the relationship between dependent and independent variables (sociodemographic, occupational and living conditions).

The multiple models were elaborated by the stepwise forward method, in which the independent variables were added individually according to the pre-determined order (decreasing values of significance). In the model, the statistically significant variables remained (p<0.05), according to the Wald test. All analyzes were adjusted for the variables gender, age, years of teaching at the masters and/or doctorate level, as pointed in the literature^(^ 2 ^,^ 17 ^)^, and senior professor and number of PPG affiliation, because we consider potential confounders of this relationship.

The Hosmer-Lemeshow and Nagelkerke R Square tests verified the quality of the final model adjustment and the explained variation of the models in relation to the outcomes, respectively. The results were presented in unadjusted and adjusted odds ratios (OR) with 95% confidence intervals.

In developing this study, the current principles of ethics in research involving human beings at the national and international levels were followed, including approval by the Research Ethics Committee of the State University of Londrina, according to Opinion no. 2.347.839.

## Results

The sample of this study was composed of 333 teachers with a median age of 53 years (IIQ: 18 years). The majority were female (87.7%), with a stable marital relationship (69.4%), with 45.6% coming from the Southeast, 26.7% from the South, 18.6% from the Northeast, 6.6% from the Midwest and 2.4% from the North.

Regarding occupational characterization, 66.7% had an exclusive work regime, 7.2% were senior professors, 19.8% received publishing grants and 29.7% were accredited to two or three programs. The median working time in PPG was, on average, 8 years (IIQ: 8 years) of teaching, weekly hours dedicated to postgraduate studies was 10 hours (IIQ: 12 hours) and hours in addition to the work contract on nights or weekends was 10 hours (IIQ: 13 hours). The professors worked at both master’s and doctoral level (70.6%), in PPGEnf with three (13.2%), four (25.2%), five (46.8%), six (6,3%) and seven (8.4%) CAPES grades.

Still, on working conditions in graduate school, 69.7% pointed they were under demand for scientific publishing, and 71.2% were satisfied with their work in graduate school. The number of graduate students and articles in progress were both in median 5 (IIQ: 4); the number of opinions issued in the last 12 months for journals or development agencies, the median was 8 (IIQ: 8).

In addition, 39.9% reported anxiety in the development of work activities, 62.5% believed that the pace and intensity of work negatively influence their lives, 47.4% stated difficulty in reconciling work with personal and family life, 72.7% thought they had enough memory and concentration ability, 63.4% stated few leisure opportunities and 56.8% were satisfied with sleep and rest.

Regarding the dimensions of DUWAS, 20.1% had great compulsive work and 19.5% high excessive work. From the combination of these dimensions, work patterns emerged, in which 70.9% were classified as positive workers, 9.0% hard workers, 9.6% compulsive workers, and 10.5% workaholics.

The multiple model presented in Table 1 showed an excellent fit (Hosmer-Lemeshow: 0.977) and explained 38.0% of the occurrence of compulsive work (Nagelkerke R Square: 0.380).

**Table 1:** Multiple model with factors associated with compulsive work among stricto sensu graduate nursing professors (n=333). Brazil, 2018

Multiple model	p-value	Odds ratio^unadjusted^ (95% confidence interval)	p-value	Odds ratio^adjusted^ * (95% confidence interval)
PPGEnf grade accredited to (3, 4 and 5 vs. 6 and 7 † )	0.003	1 0.319(0.150-0.675)	0.005	1 0.324(0.148-0.707)
Publishing grants (no vs. yes † )	0.011	1 3.210(1.300-7.930)	0.016	1 3.417(1.258-9.284)
Negative influence of work pace and intensity on life (no vs. yes † )	0.002	1 3.181(1.556-6.500)	0.001	1 3.356(1.596-7.056)
Difficulty in reconciling work with personal and family life (no vs. yes †)	0.011	1 2.875(1.278-6.467)	0.018	1 2.697(1.183-6.147)
Anxiety in the development of activities (no vs. yes † )	0.007	1 3.486(1.413-8.597)	0.007	1 3.467(1.394-8.622)
Weekly hours in addition to the regular work schedule (≤10 h vs. ≥11 h †)	0.015	1 2.475(1.193-5.135)	0.024	1 2.428(1.122-5.254)
Satisfaction with sleep (dissatisfied vs. satisfied † )	0.009	1 0.340(0.152-0.760)	0.011	1 0.342(0.149-0.783)

* adjusted for sex, age, years of teaching in a graduate program, being a senior professor, number of graduate programs to which the teacher is accredited;

† reference category vs. test category

Regarding the factors associated with compulsive work, it was found to have higher chances those professors in PPGEnf with CAPES 3, 4 and 5 grades, those who had publishing grants, those who reported the negative influence of work pace and intensity on life, those who found it more difficult to reconcile work with personal and family life, anxiety in the development of their work activities, those who claimed to work more than 11 hours a week in addition to the regular work and those who indicated they were dissatisfied with sleep.

Table 2 shows the multiple explanatory model of 35.4% of the occurrence of overwork (Nagelkerke R Square: 0.354), which is adequately adjusted (Hosmer-Lemeshow: 0.544).

**Table 2:** Multiple model with factors associated with overwork among stricto sensu graduate nursing professors (n=333). Brazil, 2018

Multiple model	*p-value*	Odds ratioun^adjusted^ (95% confidence interval)	*p-value*	Odds ratio^adjusted^ * (95% confidence interval)
Marital status (without vs. with relationship † )	0.042	1 2.195(1.030-4.678)	0.036	1 2.331(1.059-5.132)
Demand for scientific publishing (no vs. yes † )	0.029	1 2.629(1.104-6.259)	0.031	1 2.681(1.094-6.570)
Weekly hours dedicated to graduate studies (≤10 h vs. ≥11 h †)	0.004	1 0.375(0.191-0.734)	0.007	1 0.383(0.192-0.767)
Opinions issued in the last 12 months (≤20 vs. ≥21 † )	0.027	1 3.122(1.141-8.540)	0.038	1 3.014(1.062-8.548)
Articles in progress (≤10 vs. ≥11 † )	0.015	1 3.366(1.262-8.981)	0.022	1 3.198(1.187-8.622)
Satisfaction in graduate work (dissatisfied vs. satisfied † )	0.003	1 0.371(0.192-0.717)	0.006	1 0.394(0.202-0.770)
Concentration ability (little vs. much † )	0.004	1 0.371(0.190-0.723)	0.002	1 0.340(0.169-0.683)
Leisure opportunities (few vs. many † )	0.004	1 0.299(0.131-0.680)	0.002	1 0.260(0.111-0.609)
Satisfaction with sleep (dissatisfied vs. satisfied † )	0.038	1 0.488(0.248-0.959)	0.047	1 0.496(0.248-0.991)

* adjusted for sex, age, years of teaching in a graduate program, being a senior professor, number of graduate programs to which the teacher is accredited;

† reference category vs. test category

As demonstrated, professors with a stable marital relationship, who stated high-demand for scientific publishing, those who reported issuing more than 21 opinions in the last 12 months and those who reported having more than 11 articles in progress in the time of collection presented significantly increased chances of overwork. On the other hand, there were significantly reduced chances of high overwork, professors who spent less than 10 hours a week to regular graduate work, those who were satisfied with their work in graduate school, with a great ability of concentration, with many opportunities leisure and satisfied with sleep.

## Discussion

The prevalence of workaholism was 10.5% among the masters and doctoral professors studied, is lower than that obtained among hospital nurses in Italy^(^ 18 ^)^. However, the Italian study used the median as the cutoff point for the investigation, the 75^th^ percentile was used, which may be a limitation in determining the number and type of people affected by workaholism^(^ 2 ^)^.

Workaholics work excessively and compulsively, that is, they abdicate moments of leisure or social and family life due to work pace, however, in most cases, they do not achieve the desired performance, because workaholism increases the vulnerability to disability labor, due to the impairment of biopsychosocial health^(^ 19 ^-^ 20 ^)^.

The prevalence identified in this study concerning the dimensions of workaholism points that two out of ten professors dedicate an excessive amount of time to work and think persistently and continuously at work, even outside the university. It should also be considered that 7.2% of the professors in the study sample were unable to leave work at the time of retirement, since they returned to activities at PPGEnf as senior professors, even without a financial employment relationship. About this, a study carried out with professors from a Brazilian public university revealed that they return to work due to the desire to continue experiencing the feelings of pleasure that work provides, as well as the contribution they can provide due to the professional experience acquired^(^ 21 ^)^.

At some point in life, people will need to leave work, because preparation for retirement is essential, as it will contribute to the person’s empowerment and experience it to its fullest, without becoming physically or mentally ill^(^ 22 ^)^.

Regarding work patterns, a representative part of the participants in the investigation were classified as positive workers, as they indicated that they adequately reconcile personal and professional life, with little emotional stress and without giving up leisure time due to work. However, positive workers can develop dependence for work, in the long term, if exposed to unfavorable working conditions, such as excessive functions and activities, high demands and competitiveness and lack of professional valorization^(^ 23 ^)^.

Still, 9.0% of the teachers in this investigation were hard workers, because they predisposed to work hard, with high efficacy in the performance of their duties, without compromising their social life and 9.6% were classified as compulsive, who are dependent on work for their personal satisfaction, extrapolate the daily workload, take work home and think about work, constantly. Both profiles are indicated by the literature as predictors for workaholism since they emerge from the dimensions of excessive and compulsive work^(^ 24 ^)^.

Compulsive work, the cognitive dimension of workaholism, reflects persistent thoughts about work and constant concern with issues in the work environment, even outside professional situations^(^ 24 ^)^. The data in this study showed that high compulsive work was higher among teachers who affiliated to the PPGEnf CAPES grade 3, 4 and 5, those who received publishing grants, those who worked more than 11 hours a week in addition to the regular work, those who reported anxiety in the development of their activities, the negative influence of work pace and intensity on their lives, those who felt greater difficulty in reconciling work with personal and family life and those dissatisfied with sleep.

It is believed that the PPGEnf of lower grades in the ranking by CAPES want to reach better grades, because CAPES grades 6 and 7 represent excellence in the training of professor-researchers^(^ 10 ^)^ and, consequently, implies raising more financial resources. In this way, PPGEnf teachers with a lower CAPES grade may feel compelled to work harder to achieve better publishing indicators, since the qualification of the program depends on the professor’s engagement. This causes this professional to keep constant thinking and concerns about labor issues, focused on achieving the stipulated goals, thus replacing his moments of leisure and rest with work outside the university.

Teachers who received a publishing grant had more chances of high compulsive work, which is awarded to researchers, leaders, and paradigms in their areas of knowledge, classifying them in levels, according to their scientific, technological and innovation publishing. In addition to being a way of recognition and appreciation, the granting of the scholarship aims to encourage an increase in qualified publishing^(^ 25 ^)^. The findings of this study indicated that teachers with this benefit had an addictive relationship with work, which may be an attempt to maintain the status of a reference researcher in their area of expertise.

Professors in this study who worked more than 11 hours a week in addition to the regular work, on nights and weekends also had a greater chance of high compulsive work. Such a result may be related to the need to perform multiple activities in a short time, especially those that need theoretical deepening.

The overload of activities causes the teacher to get used to the intense pace of work, not being able to disconnect from his/her professional duties mentally, feel guilty for resting considering the numerous pending activities or get frustrated, for feeling useful only when working hard^(^ 19 ^)^. Thus, he/she is unable to manage time properly, as he/she is anxious to develop his/her work activities, even though he/she realizes that the pace and intensity of work are negatively influencing his life and remain to relegate his/her personal and social life, leisure and sleep because he/she believes that work alone brings professional and own satisfaction.

The behavioral dimension of workaholism is overwork and is manifested by hard work, in which the person spends a large amount of time on work activities, working above their economic needs and the demands of the organizations to which they belong^(^ 24 ^)^. Significantly increased chances of high overwork occurred among professors with a domestic relationship, who feel greater pressure for scientific publishing, those who issued more than 21 opinions in the last 12 months, those who have more than 11 articles in progress, those who dedicate less 10 hours a week to graduate school, those dissatisfied with their work in graduate school, with little ability to concentrate, few leisure opportunities and dissatisfied with sleep.

Regarding the association between a stable marital relationship and overwork, the literature explains only about the unsatisfactory family relationship and bad marriage, that is, the one that is considered as conflictual or that does not bring satisfaction, and can make people work long hours^(^ 26 ^)^.

Significantly greater chances of high overwork were found among the professors who participated in this research due to pressure for scientific publishing, issuing many opinions annually and having many articles in progress. Publication in scientific issues represents the dissemination of knowledge and has been widely used for teacher evaluations (career advancement, qualification of the program, promotion of research, obtaining scientific initiation and publishing grants, among others). However, preparing articles for specialized and well-qualified journals requires knowledge, investment of time, dedication, focus and discipline, so that they have the quality, relevance and novelty, that is, that contribute to the advancement of knowledge^(^ 27 ^-^ 28 ^)^.

Issuing opinion on a scientific article also requires time, considering the responsibility to critically and constructively analyze the originality, the coherence between theoretical, methodological and interpretations, to collaborate with the improvement of the research in question.

It is inferred that excessive work among graduate professors is related to intense mental activity, in an attempt to achieve expectations promptly, given the quantity and complexity of tasks, which occur besides to other activities. This situation can also lead to dissatisfaction with work in graduate school.

The relationship between overwork and dissatisfaction with sleep can derive from nights, late nights, and moments of rest relegated due to work activities, disrupting the sleep-wake cycle, especially in the quality of sleep and lasting^(^ 29 ^)^. It should be noted that most teaching activities, due to their diversity, are challenging to measure in hours and are not usually included in the weekly workload. However, they become mandatory due to their roles and goals established by the programs.

The study has a limitation because it was carried out with a self-applied questionnaire and, consequently, the possibility of biased responses, considering that the condition of workaholic leads the individual to self-denial, with attempts to fit his condition with an committed worker^(^ 30 ^)^. However, it has potentialities such as the expressive sample, distributed in all regions of the country, associating the working conditions of nursing professors from PPGEnf to a phenomenon little studied in the area. Workaholism is a problem of a subjective nature and, therefore, can be influenced by personal characteristics and the work process can contribute to its emergence or aggravation, since producing more and more is the motto of the capitalist world. Thus, it is suggested that other studies include the mediating effect of personality to deepen the theme.

Finally, the current Brazilian socio-political situation has contributed to the deterioration of working conditions, especially those related to higher education and scientific research. The results of this research reflect a system of high demands and competitiveness, permeated by the scarcity of financial resources and infrastructure, in which, teachers invest their time and health to train staff and advance knowledge.

## Conclusion

The results allow us to conclude that there is a sign of workaholism in the studied sample and it was identified as associated factors: domestic relationship, as a sociodemographic aspect; living conditions: dissatisfaction with sleep, reduced ability to concentrate and few leisure opportunities; and teaching work conditions: being accredited to the PPGEnf CAPES grade 3, 4 and 5, being a publication grant, demand for scientific publication, dissatisfaction with postgraduate work, performing more than 11 hours a week in addition to the regular work, dedicating less than 10 hours a week to graduate school, issuing many opinions in a short period of time, write many articles simultaneously, the negative influence of work pace and intensity on life, difficulty in reconciling work with personal and family life and anxiety in the development of activities labor.

These findings indicate the characteristics and requirements of the master’s and doctoral professors’ work process, positively and directly associated with workaholism, which points to the importance of professors along with managers, to review the quality criteria based on quantity, since they are the government agencies that set the parameters. It is still crucial that institutions adopt management models with policies of emotional support and well-being, in addition to providing adequate working conditions for their teachers, envisioning occupational health.
